# A Less Saline Baltic Sea Promotes Cyanobacterial Growth, Hampers Intracellular Microcystin Production, and Leads to Strain-Specific Differences in Allelopathy

**DOI:** 10.1371/journal.pone.0128904

**Published:** 2015-06-04

**Authors:** Andreas Brutemark, Angélique Vandelannoote, Jonna Engström-Öst, Sanna Suikkanen

**Affiliations:** 1 ARONIA Coastal Zone Research Team, Novia University of Applied Sciences & Åbo Akademi University, Ekenäs, Finland; 2 Tvärminne Zoological Station, University of Helsinki, Hanko, Finland; 3 Marine Research Centre, Finnish Environment Institute SYKE, Helsinki, Finland; INRA, FRANCE

## Abstract

Salinity is one of the main factors that explain the distribution of species in the Baltic Sea. Increased precipitation and consequent increase in freshwater inflow is predicted to decrease salinity in some areas of the Baltic Sea. Clearly such changes may have profound effects on the organisms living there. Here we investigate the response of the commonly occurring cyanobacterium *Dolichospermum* spp. to three salinities, 0, 3 and 6. For the three strains tested we recorded growth, intracellular toxicity (microcystin) and allelopathic properties. We show that *Dolichospermum* can grow in all the three salinities tested with highest growth rates in the lowest salinity. All strains showed allelopathic potential and it differed significantly between strains and salinities, but was highest in the intermediate salinity and lowest in freshwater. Intracellular toxin concentration was highest in salinity 6. In addition, based on monitoring data from the northern Baltic Proper and the Gulf of Finland, we show that salinity has decreased, while *Dolichospermum* spp. biomass has increased between 1979 and 2013. Thus, based on our experimental findings it is evident that salinity plays a large role in *Dolichospermum* growth, allelopathic properties and toxicity. In combination with our long-term data analyses, we conclude that decreasing salinity is likely to result in a more favourable environment for *Dolichospermum* spp. in some areas of the Baltic Sea.

## Introduction

The Baltic Sea is one of the largest brackish water basins on earth and has a wide range in salinity. Surface salinity decreases from south to north and varies from ~23 in the Kattegat, 9 in southern Baltic Proper to <1 in the eastern parts of the Gulf of Finland and the northern parts of the Bothnian Bay. It is expected that river runoff and precipitation will increase with climate change in the drainage area, and thereby increase the freshwater inflow to the Baltic Sea [1,2]. Further, there is a substantial annual variation in the amount of saline water entering the Baltic Sea [3]. As a result of the combined effects of decreasing inflow of saline water into the Baltic Sea via the Danish Straits, and increasing freshwater inputs, a decreasing salinity trend has been observed for some Baltic Sea basins [4,5,6,7].

The summer in the Baltic Sea is characterized by cyanobacterial mass occurrences, consisting mainly of three filamentous diazotrophic taxa: *Aphanizomenon* sp., *Nodularia spumigena* and *Dolichospermum* spp. (ex. *Anabaena* spp. [8]). *N*. *spumigena* produces the hepatotoxin nodularin that affects aquatic organisms, causes poisonings of domestic animals as well as wildlife, and can pose a hazard for human health [9,10]. *Aphanizomenon* sp. and *Dolichospermum* spp. are known to produce toxins in freshwaters [11] and more recently, *Dolichospermum* has occasionally proven to be hepatotoxic in the Baltic Sea, producing microcystins [12,13].

The role of toxins is debated, but the production of chemical compounds to kill or limit growth of competitors may be of competitive advantage to phytoplankton under certain environmental conditions [14]. Allelopathy, defined by the International Allelopathy Society (as in [15]) as”any process involving secondary metabolites produced by plants, microorganisms, viruses and fungi that influence the growth and development of agricultural and biological systems”, seems to be common for many groups of phytoplankton, including chlorophytes, diatoms, dinoflagellates, haptophytes, raphidophytes, and cyanobacteria (see reviews by [14,15]). Both biotic and abiotic factors, including the species involved and their density, growth phase, nutrient concentrations, pH, light intensity, and temperature have been suggested to affect the allelopathic potency (reviewed by [15]).

The role of salinity in the production of cyanobacterial toxins and/or allelopathic compounds is largely unknown. The levels of nodularin increased with increasing salinity for the Baltic *N*. *spumigena* [16], whereas Blackburn et al. [17] found an opposite effect; toxin content decreased in Australian *N*. *spumigena* with rising salinity. In the experiment by Lehtimäki et al. [18], *N*. *spumigena* toxin levels were highest in the salinity range 5–20 compared with higher and lower salinities. Further, Halinen et al. [12] suggested that salinity was the best explanatory factor for the presence of microcystin-producing *Dolichospermum* sp. in the Gulf of Finland. Toxic *Dolichospermum* sp. was only found in environments with low salinity [12], indicating that microcystin production was favoured in low salinities (cf. [19]). As some basins of the Baltic Sea are becoming less saline [4,5,6,7,20], we can consequently expect a change in toxicity of the phytoplankton present. Since *Dolichospermum* spp. are common freshwater species, a reduced salinity can promote a competitive advantage. Thus salinity can play a large role in growth and toxin production and overall fitness of *Dolichospermum* spp. Indeed, the northern Baltic Proper has over the last 34 years become less saline, while the biomass of cyanobacteria has increased [7]. However, little information is available concerning the allelopathic capabilities in this changing environment. The aim of this study is therefore to determine the effect of salinity on *Dolichospermum* spp. growth, intracellular microcystin concentrations and allelopathic properties towards a co-occurring non-toxic cryptophyte *Rhinomonas nottbecki*. We used salinities relevant for the Gulf of Finland—northern Baltic Proper, i.e., stretching from a freshwater environment (salinity 0), via an intermediate salinity (3), to brackish waters (6). In addition, by using long-term monitoring data for western Gulf of Finland and northern Baltic Proper, we addressed salinity and *Dolichospermum* spp. biomass trends from 1979 to 2013.

## Materials and Methods

### Ethics statement

No permits were required for the described study, and the study did not involve endangered or protected species.

### Strains and cultivation

For the experiment, three different strains of *Dolichospermum* sp. were used: BIR250A, BIR256 and BIR257, isolated by Katrianna Halinen from the Gulf of Finland, Baltic Sea in 2004, and provided by Prof. Kaarina Sivonen (University of Helsinki). Prior to the experiment the strains were cultured for more than one year under the targeted salinity conditions (salinity 0, 3 and 6) as batch cultures at 18°C, in a 16:8 h light:dark cycle with a ~8 μmol photons m^-2^ s^-1^ light intensity using Z8 medium [21] with nitrogen omitted and NaCl added to reach the desired salinities. Salinity was continuously measured using a refractometer as well as a conductivity meter (EC300 VWR).

The cryptophyte *R*. *nottbecki* strain 07B6 (isolated from the Baltic Sea in 2007 by Dr. Anke Kremp) was grown in F/2 medium with silicate omitted [22] at 16°C and salinity 6.

### Experimental design

For the growth experiment, each *Dolichospermum* sp. strain was cultivated in triplicate 300 mL tissue culture flasks (total culture volume 200 mL) at 18°C, in 35 μmol photons m^-2^ s^-1^ in a 14:10 h light:dark cycle. The batch culture experiment was started from a cell concentration of 70 000 cells mL^-1^ and growth and toxicity of the cultures were monitored for 29 days.

In mid-exponential phase (day 18–20) as well as late stationary phase (day 43), a separate experiment was conducted to study allelopathic effects of cell-free filtrates of the strains on the growth of the cryptophyte *R*. *nottbecki*. Allelopathic effects were expressed as EC_50_, i.e., the *Dolichospermum* cell concentration yielding a 50% decline in *R*. *nottbecki* fluorescence (modified from [23]). Filtrates of each of the *Dolichospermum* sp. strains in the different salinities were prepared using 2.0 μm polycarbonate filters (Whatman, Nuclepore). The filtrate salinity was adjusted to 6 using NaCl, as *R*. *nottbecki* was grown in this salinity. F/2-Si medium nutrients were added to both the control Z8 medium and the filtrates in order to provide excess nutrients for *R*. *nottbecki*. In addition, pH of the filtrates was measured (using a Metrohm 780 pH meter, calibrated with Merck CertiPUR 4.01 and 7.00 buffers) and a 1.5 mL sample of each filtrate was collected and frozen until toxin analysis.

A total of seven *Dolichospermum* sp. filtrate dilutions and a control were prepared ranging from 0 to 366 700 cells mL^-1^ in exponential phase, and 0 to 1 000 000 cells mL^-1^ in stationary phase (maximum number of cells was determined by the least dense culture). To dilute the filtrate, Z8 medium with nitrogen omitted (salinity 6) was used. The EC_50_ experiments were conducted in 12-well culture plates. Control medium and each *Dolichospermum* filtrate dilution were added in triplicate to a fixed initial *R*. *nottbecki* cell density (10 000 cells mL^-1^). The total experimental volume in one well was 4 mL (3.9 mL *Dolichospermum* sp. filtrate dilution or control Z8 medium, 0.1 mL *R*. *nottbecki* cell suspension). The plates were incubated for 4 days at 18°C in 80 μmol photons m^-2^ s^-1^ light before fluorescence was measured as described below.

### Analytical procedures

To identify an optimal *Dolichospermum* sp. growth phase for the allelopathy experiments, the growth of the strains was followed on an almost daily basis (1–2 day intervals) by measuring chlorophyll *a* fluorescence (data not shown). The fluorescence was read from 400 μL samples in a 96-well plate with Varian Cary Eclipse Fluorescence Spectrophotometer, using excitation wavelength 625 nm and emission wavelength 683 nm [24]. In the EC_50_ experiment, fluorescence of *R*. *nottbecki* was measured after 4 days (Ex. 440 nm, Em. 680 nm).

Cell count samples (1.2 mL) for *Dolichospermum* sp. were collected and preserved with a drop of acid Lugol’s solution and stored in Eppendorf tubes in a refrigerator. The cell counts were performed using a 1 mL Gridded Sedgewick Rafter (Wildlife Supply Company) counting chamber and a microscope (Leica DMI3000 B) with a 200 × magnification. From each *Dolichospermum* sp. sample, the cells of at least 50 filaments were counted. Cell width and length were measured for each strain and salinity (N = 20), and biovolume was determined [25]. Growth rates, *k*, defined as doublings d^-1^, were calculated based on the longest period of exponential growth, using the equation *k* = log_2_(N_t_/N_0_)/Δt, where *N* = biomass and *t* = time [26]. The interval of exponential growth was determined from growth curves established for each experimental culture replicate.

Both intracellular and extracellular toxins (for the allelopathy experiment) were measured using ELISA (Enzyme-linked immunosorbent assay) QuantiPlate Kit for Microcystin (Envirologix) following the kit instructions. Ten mL samples were taken from the cultures on three sampling days (day 1, 15 and 29) and filtered using 2.0 μm TTTP membrane filters (Isopore, Millipore). Both the filter (containing *Dolichospermum* sp. cells, i.e., intracellular toxins) and the 1.5 mL filtrate samples from the allelopathy experiment (i.e., extracellular toxins) were stored in -20°C. Before analysis, the filters were freeze-dried for at least 48 h. The intracellular samples were dissolved in 5 mL methanol, sonicated for 5 min and extracted overnight. The samples were dried using compressed air and dissolved again by adding 100 μL 50% methanol, followed by adding 900 μL of MilliQ over 4 days (225 μL MilliQ d^-1^). On the final day of MilliQ addition (final concentration of 5% methanol [27]), the samples were measured. The intracellular toxin samples were diluted (1:20–1:1000) and filtered on Whatman GF/C filters before performing ELISA. The extracellular samples were completely melted and shaken properly before measurement. The microcystin concentration was determined using a micro-plate reader (Tecan, Infinite M200) and was based on a standard curve (standards: 0.16, 0.6 and 2.5 ppb).

### Statistical analyses

To analyse differences in biovolume, growth rate and allelopathic effect between strains, salinities and growth phases, a general linear model (GLM) was fitted separately to each response variable. The explanatory variables for biovolume and growth rate included strain and salinity and their interaction. A separate GLM was fitted to check the influence of strain, salinity and growth rate and their interactions on exponential phase (day 15) intracellular toxicity. For allelopathic effect as response variable, a GLM with main effects for strain, salinity and growth phase, and interactions between strain and growth phase, and salinity and growth phase was selected based on the model’s Akaike information criterion (AIC) value. Percentage reduction of *R*. *nottbecki* fluorescence by filtrates from both growth phases prepared from a concentration of ca. 4–5×10^5^ cells mL^-1^ was used as a measure of allelopathic potency.

Due to temporal dependence of toxin measurements conducted at 3 time points during the experiment, a linear mixed-effects (LME) model (R package ‘nlme’ [28]) was used to analyse differences in intracellular toxin concentrations between salinities, days and strains, with salinity and day as fixed effects and strain as a random effect. Statistical significance of the random effect for strain was assessed by comparing a generalized least squares (GLS) model without random effect, but with salinity and day as main effects, with the final LME model. The comparison was executed by using a likelihood ratio test (LRT). Distributional assumptions of linear models (normality and homoscedasticity of residuals) were checked for each response variable, and biovolume and toxin data were log-transformed before analysis to meet the assumptions. Tukey’s pairwise post-hoc comparisons (R package ‘lsmeans’ [29]) were used to determine which of the effects of explanatory factors and their interactions differed significantly from each other.

The EC_50_ (effective dose) value was calculated from the best fitting dose-response model (judged by the smallest AIC value), using the R package ‘drc’ [30].

The non-parametric Spearman’s rank correlation was used to assess the degree of correlation between toxin concentration and allelopathic effects of the filtrates.

To check for decadal trends in salinity and *Dolichospermum* spp. biomass, monitoring data from 7 sampling stations in the western Gulf of Finland—northern Baltic Proper (sampling area with spatial limits N 59°03–59°85, E 21°08–24°83) between 1979 and 2013 were analysed. The data originated from national monitoring cruises, where sampling is conducted according to the Helsinki Commission (HELCOM) COMBINE programme, and they were downloaded from the databases Sumppu (Finnish Environment Institute and Finnish Meteorological Institute; nodc.fmi.fi/grafeio) and Hertta (Finnish Environment Institute; wwwp2.ymparisto.fi/scripts/oiva.asp). A non-parametric Mann-Kendall test was used to detect significant monotonic trends in the annual mean summer-early autumn (June-September) salinity and *Dolichospermum* spp. biomass at 0–10 m depth. Curves estimated with a locally weighted scatterplot smoother (LOESS; span = 0.75) with 95% confidence interval were fitted to describe the long-term variation.

All analyses were conducted using R 2.15.2 software [31].

## Results

### Biovolume and growth

In the batch-culture experiments, *Dolichospermum* spp. cell size differed significantly between strains and salinities, varying between 58.97 ± 14.68 μm^3^ cell^-1^ (mean ± SD, n = 20) in BIR257 at salinity 6 and 159.58 ± 45.13 μm^3^ cell^-1^ in BIR256 at salinity 3 (GLM, p < 0.01, Tables 1 and 2). On average, BIR257 had a smaller biovolume than the other two strains (Tukey, p < 0.04), and the cells of all strains were largest at salinity 3 and smallest at 6 (Tukey, p < 0.001).

**Table 1:** Mean cellular biovolume (V; n = 20), and filtrate pH, toxin concentration and EC50 value (n = 3) of the *Dolichospermum* spp. strains in different salinities and growth phases.

				Exponential phase					Stationary phase		
Strain	Salinity	V μm^3^ cell^-1^	SD_V_	pH	Toxin fg cell^-1^	SD_tox_	EC_50_ cells ml^-1^	SE_EC50_	pH	EC_50_ cells ml^-1^	SE_EC50_
BIR250A	0	109.18	28.97	7.93	6.97	0.81	208317	22370	7.45	NA	NA
BIR250A	3	123.43	81.40	7.35	2.48	0.52	264321	>1.5*106	7.40	407347	132259
BIR250A	6	72.58	25.83	7.36	11.00	9.43	544421	106765	7.27	>2.4*106	>1.0*106
BIR256	0	84.76	51.64	7.56	134.64	7.41	>2.5*106	>2.3*106	7.62	>1.8*106	395316
BIR256	3	159.58	45.13	7.28	0.66	0.02	62070	21458	7.27	>1.1*106	114792
BIR256	6	60.51	23.11	7.29	0.54	0.04	>1.0*106	708327	7.45	153061	109919
BIR257	0	66.32	12.67	7.37	180.65	38.11	NA	NA	7.37	NA	NA
BIR257	3	106.91	38.57	7.15	0.50	0.10	124458	338876	7.31	NA	NA
BIR257	6	58.97	14.68	7.26	2.12	0.51	NA	NA	7.28	228698	96346

NA = not available, effect either positive or non-quantifiable.

**Table 2:** Results of general linear models (GLM) and linear mixed-effects (LME) models for each response variable.

Model type	Response variable	Explanatory variable	*F*	*p*
GLM	Biovolume	Strain	4.90	0.009
GLM	Biovolume	Salinity	38.47	<0.001
GLM	Biovolume	Strain*Salinity	6.14	<0.001
GLM	Growth rate	Strain	19.75	<0.001
GLM	Growth rate	Salinity	24.65	<0.001
GLM	Growth rate	Strain*Salinity	3.14	0.040
LME	IC toxin concentration	Salinity	12.51	<0.001
LME	IC toxin concentration	Day	8.28	<0.001
LME	IC toxin concentration	Strain	NA	0.277
GLM	IC toxin concentration (exp. phase)	Strain	0.87	0.441
GLM	IC toxin concentration (exp. phase)	Salinity	14.73	<0.001
GLM	IC toxin concentration (exp. phase)	Growth rate	0.38	0.549
GLM	IC toxin concentration (exp. phase)	Strain*Salinity	1.54	0.240
GLM	IC toxin concentration (exp. phase)	Growth rate*Salinity	4.41	0.031
GLM	Allelopathic effect	Strain	5.62	0.007
GLM	Allelopathic effect	Salinity	9.35	<0.001
GLM	Allelopathic effect	Growth phase	0.02	0.880
GLM	Allelopathic effect	Strain*Growth phase	8.13	<0.001
GLM	Allelopathic effect	Salinity*Growth phase	14.88	<0.001

For the LME models with salinity and day as fixed factors and strain as a random factor, significance of strain was derived from Likelihood ratio test.

**Fig 1:**
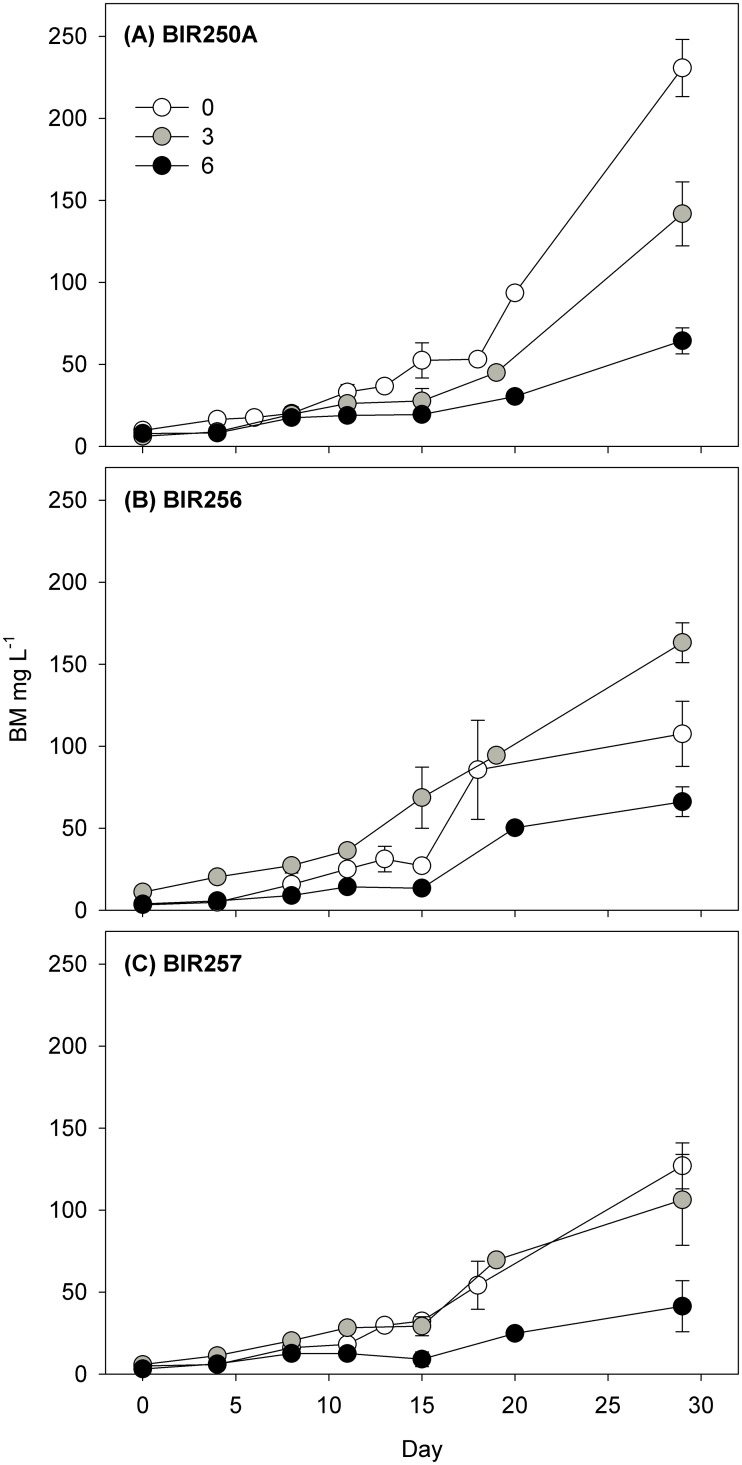
Biomass development of the *Dolichospermum* spp. strains in the experimental salinities (mean ± SD, n = 3).

**Fig 2:**
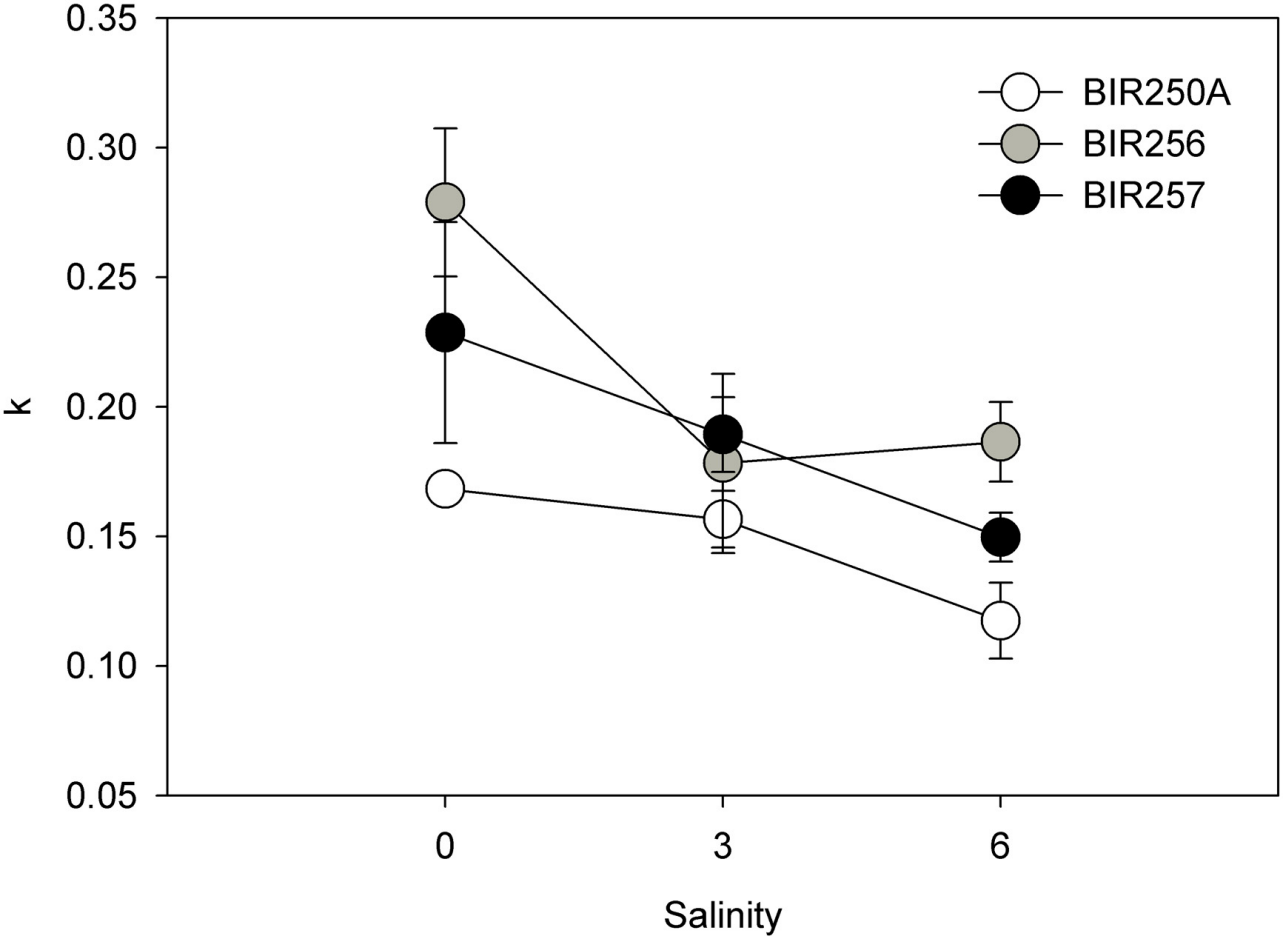
Biomass based growth rates (k, doublings day-1) of strains in the different salinities (mean ± SD, n = 3).

Growth rates of the strains were also significantly dependent on the strain and salinity, ranging from 0.12 ± 0.01 doublings per day (mean ± SD, n = 3) in BIR250A at salinity 6 to 0.28 ± 0.03 doublings day^-1^ in BIR256 in freshwater (GLM, p < 0.04, Table 2, Figs 1 and 2). Although BIR250A reached the highest observed final biomass in freshwater (Fig 1), the strain had, on average the lowest maximum growth rates (Tukey, p < 0.004, Fig 2). Growth rates of all strains were highest at salinity 0 (p < 0.001).

### Microcystin concentrations

**Fig 3:**
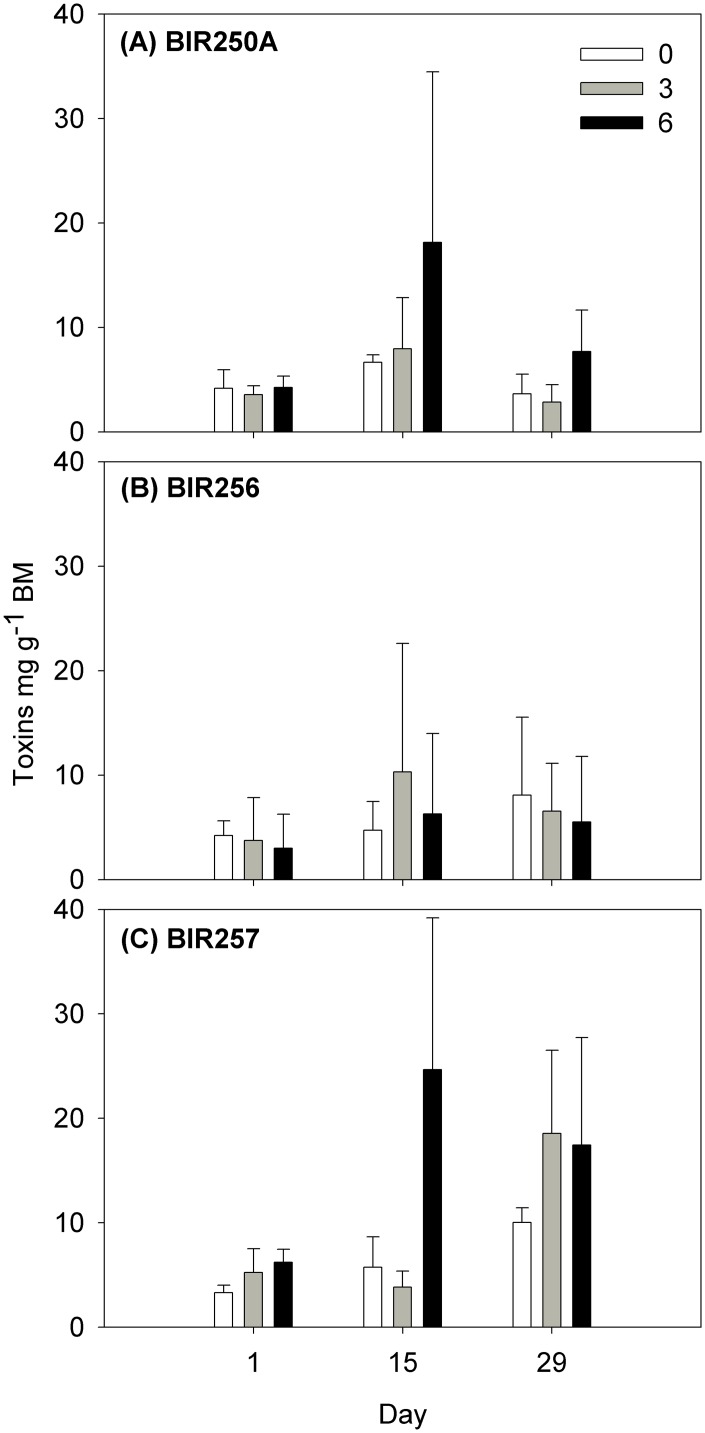
*Dolichospermum* spp. intracellular microcystin concentrations (mean ± SD, n = 3) in different salinities at the start, middle and end of the experiment.

The intracellular microcystin concentrations of the *Dolichospermum* sp. cultures differed significantly between salinities and days (LME, p < 0.001; Table 2, Fig 3), but not between strains (p = 0.277). The mean intracellular microcystin concentration ranged from 1.70 ± 1.34 mg g BM^-1^ (mean ± SD, n = 3) in BIR256, salinity 3 on day 1, to 24.66 ± 14.54 mg g BM^-1^ in BIR257, salinity 6 on day 15, and was for all strains on average highest at salinity 6 and lowest on day 1 (Tukey p < 0.03, Fig 3). In addition, we tested for a potential effect of growth rate, strain and salinity on intracellular toxin concentrations during exponential growth. In addition to a significant effect of salinity (GLM, p < 0.001), but no direct effects by strain or growth rate (p > 0.40), there was a significant interaction effect between growth rate and salinity (p = 0.031; Table 2), indicating that the growth rate may have affected toxicity differently in the different salinities.

### Allelopathic effects

The pH of the *Dolichospermum* sp. filtrates, used for testing their allelopathic properties, varied from 7.15 to 7.93 (Table 1), whereas the average pH of the *R*. *nottbecki* culture was 9.74 and that of the control medium (Z8, salinity 6) 7.55.

**Fig 4:**
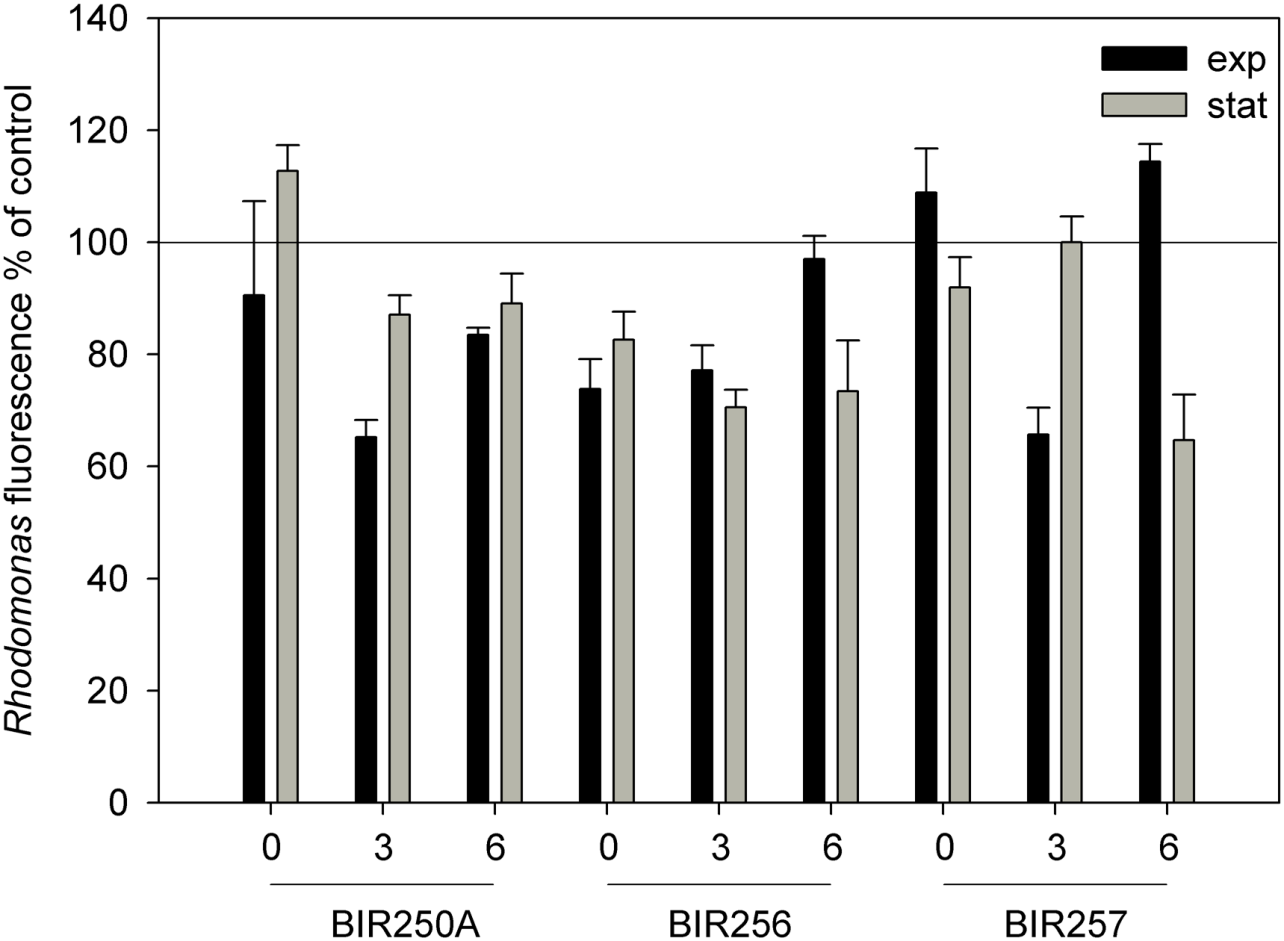
Allelopathic effect of *Dolichospermum* spp. on *R*. *nottbecki*. Expressed as percentage fluorescence of *R*. *nottbecki*, cultured for 4 days in filtrates of exponential and stationary phase *Dolichospermum* spp. cultures (cell concentrations 4–5×10^6^ mL^-1^), in relation to control (100%) (mean ± SD, n = 3).

All strains showed allelopathic potential by reducing the fluorescence of *R*. *nottbecki* during 4 days (Table 1, Fig 4), although during some conditions, the effects were undetected or even positive. With an EC_50_ value of ca. 6.2×10^4^ cells mL^-1^, the exponential phase filtrate of BIR256 at salinity 3 was the most effective one against *R*. *nottbecki* (Table 1). Percentage reduction of *R*. *nottbecki* fluorescence by filtrates prepared from a comparable cell concentration (Fig 4) generally reflected the EC_50_ values, but the most effective strain, based on this approach, was the stationary phase BIR257 strain at salinity 6, causing a 35.4 ± 8.2% (mean ± SD, n = 3) reduction in *R*. *nottbecki* fluorescence compared with the control.

Allelopathic potency differed significantly between strains and salinities (GLM, p < 0.007; Fig 4), but not between growth phases (p = 0.880; Table 2). However, interactions between strain and growth phase, as well as salinity and growth phase were significant (p < 0.001, Table 2), indicating that strain and salinity affected the allelopathic properties differently in each growth phase (see Fig 4). A significantly stronger allelopathic effect of *Dolichospermum* was observed when grown in salinity 3 and 6, for exponential and stationary phase respectively, than in freshwater (Tukey, p < 0.006). On average, BIR256 was more allelopathic than the other strains (p < 0.05), and the allelopathic effects were most pronounced at salinity 3 (p < 0.04).

The extracellular toxin concentrations in the exponential phase filtrates (expressed as toxin cell^-1^ in the original culture) ranged from 0.50 fg cell^-1^ in BIR257 in salinity 3 to 180.65 fg cell^-1^ in BIR257 in freshwater (Table 1). Allelopathic effects (percentage *R*. *nottbecki* fluorescence reduction or EC_50_ value) of the exponential phase filtrates did not correlate with their extracellular toxin concentration (Spearman’s rank correlation, r_s_ < 0.19).

### Long-term trends

**Fig 5:**
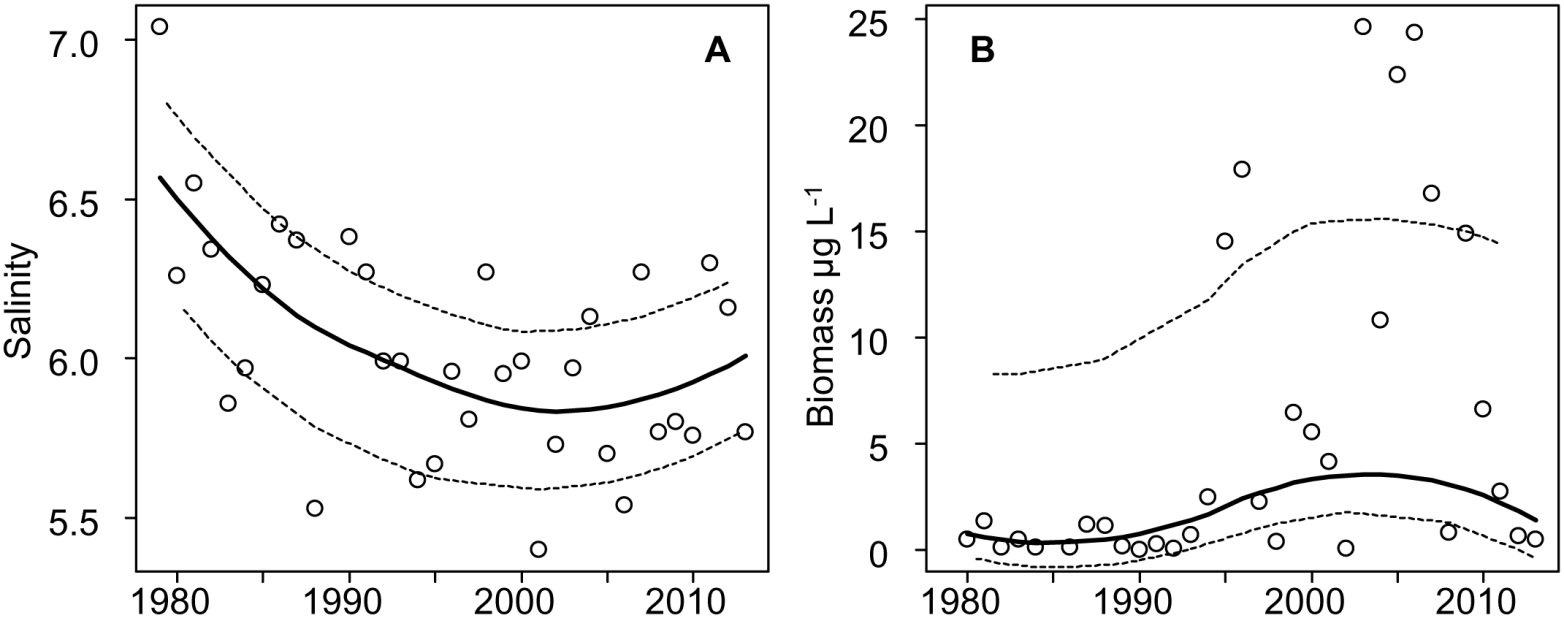
Annual mean summer-early autumn (A) salinity and (B) *Dolichospermum* spp. biomass in the western Gulf of Finland—northern Baltic proper at 0–10 m depth in 1979–2013. A Loess curve (span = 0.75; solid line) is fitted, with a 95% confidence interval (dashed line).

There was a significant decreasing trend in surface water salinity of the western Gulf of Finland—northern Baltic Proper between 1979 and 2013 (Mann-Kendall test, S = -165, N = 33, p = 0.015). During the same time span, biomass of *Dolichospermum* spp. increased significantly (S = 170, N = 33, p = 0.009). Both trends were strongest until around 2005, after which the decline in salinity and rise in *Dolichospermum* spp. biomass levelled out (Fig 5).

## Discussion

In short, this study shows that the Baltic cyanobacteria *Dolichospermum* spp. can grow in all studied salinities (0, 3 and 6), with the highest growth rate in freshwater. Highest intracellular microcystin concentration was measured in the highest salinity (6). Allelopathic properties were evident in all strains tested, and they were most pronounced at salinity 3. However, interactions between strain and growth phase, as well as salinity and growth phase were significant, indicating that strain and salinity affected the allelopathic properties differently in each growth phase. Monitoring data show that summertime surface water salinity has decreased between the years 1979 and 2013 while *Dolichospermum* spp. biomass has increased. Overall, our laboratory experiment, combined with long-term monitoring data, suggests that a decrease in salinity results in a more favourable environment for *Dolichospermum* spp.

The growth rates of the different *Dolichospermum* strains were clearly affected by salinity. Although some strain-specific variation occurred, they showed best growth in freshwater. BIR256 biomass was highest in salinity 3 during the last ten days of the experiment. The *Dolichospermum* taxa originate from freshwater and grow usually better in freshwater or low saline water, whereas some strains are adapted to brackish water (cf. [32]). *Dolichospermum* spp. are generally considered sensitive to salt, and growth decreases strongly above 10 g NaCl L^-1^ [33]. When exposed to salt stress, cyanobacteria increase the N-fixation leading to a further energy demand, and consequently a reduction in their growth rates can be detected [33]. Indeed, in the southern Baltic Sea, where salinity is higher (~10) the abundance of *Dolichospermum* is negligible [34]. Thus, it seems that Baltic *Dolichospermum* spp. can grow in salinities up to ~10 but, as we show here, they proliferate in freshwater.

Interestingly, cells of all strains were largest at salinity 3 and smallest at 6. Also Tonk et al. [35] showed that cell size of freshwater cyanobacteria decreases in increasing salinity. The authors claim that this occurs when osmoregulation capacity is exceeded, and cells no longer are able to uphold turgor, which can cause cell leakage and shrinking, when salinity rises even further. The phenomenon is called plasmolysis and is shown also in picocyanobacteria *Synechocystis* [36] and bacteria *Escherichia coli* [37].

A positive relationship between growth rate and microcystin production has been shown [38,39]. In the current study, however, the relationship between intracellular microcystin content and growth was not that clear (Table 2). It seems that growth rate affected toxin levels differently in different salinities and, thus, no direct relationship between growth and toxin levels is evident. The role of microcystins has not yet been determined but several hypotheses for toxin production among cyanobacteria have been suggested including grazer avoidance, allelopathy, infochemicals and stress reduction (cf. [40,41,42]). For example, higher microcystin production of *Microcystis* during stress has been reported in elevated temperature and during low light [43,44]. Zilliges et al. [43] found a clear fitness advantage for microcystin-producing strains, in comparison to non-producers. It is therefore possible that microcystins serve as a defence mechanism against oxidative stress. Salinity may cause oxidative stress in cyanobacteria as shown for *Anabaena doliolum*, *Fremyella diplosiphon* and *Nostoc muscorum* [45,46,47]. In the current study, one may speculate that enhanced toxin production is a result of elevated oxidative stress in high salinity environment, for some strains of this cyanobacterium having a freshwater origin. In the field, however, toxic *Dolichospermum* sp. has so far only been found in the Baltic Sea areas with salinity <6 [12]. More recently, using a microcystin-producing wild-type strain of *Microcystis aeruginosa* and a microcystin deficient-mutant, Makower et al. [48] found differences in gene expression between the two strains. The authors suggested that changes to the photosystem as well as a more pronounced carbon limitation for the microcystin deficient-mutant occurs when grown under similar conditions [48]. Salinity has been showed to induce stress responses in cyanobacteria including inhibition of photosynthesis associated genes and upregulation of genes associated with photorespiration [49]. Thus, it is possible that microcystin plays a role in salinity-induced changes of photosystems as well as photorespiration. However, to understand the underlying mechanisms of cyanobacteria responses to different salinities, further investigations are needed.

All three *Dolichospermum* strains showed allelopathic potential, which did not differ between exponential and stationary growth phase, implying that allelochemicals are continuously excreted from the cells. *Dolichospermum* has frequently been identified as allelopathic (reviewed by [50,51]). From a general perspective, allelopathy was strongest in salinity 3 and the highest allelopathic ability upon *Rhinomonas* was shown by strain BIR256. However, interactions between strain and growth phase, as well as salinity and growth phase suggest that the allelopathic capacity of *Dolichospermum* does not respond solely to changes in salinity, and that there seem to be strain-specific differences that are challenging to predict. Interestingly, a significantly stronger allelopathic effect of *Dolichospermum* is observed for exponential and stationary phase when grown in salinity 3 and 6, respectively, than when grown in freshwater. Thus, it seems as if the allelopathic potency is relatively lower in freshwater than in the two salinity (3 and 6) treatments. This suggests that salinity or salinity induced stress stimulates allelopathy in *Dolichospermum* in order to weaken competitors or otherwise harm them. Also during other stressful conditions, such as nutrient and light limitation, *Anabaena* sp. out-competed the competitor via allelopathy, and another taxon, *Oscillatoria* increased the production of allelopathic compounds [52,53]. Higher allelopathic potency allows the species to outcompete other bacterial and micro-algal competitors, such as the cryptophyte *Rhinomonas*. *Dolichospermum* is known to have strong allelopathic potency against cryptophytes both in mono-cultures and the natural community, decreasing their cell numbers by 50–80% [54,55]. Allelopathy can contribute to cyanobacterial bloom maintenance as the growth of competitors will be inhibited or competing species even exterminated. According to long-term data analyses, cryptophytes have rapidly decreased in the Baltic Sea [6,7], but the phenomenon is assumed to be associated with warming [56]. Biotic interactions, such as allelopathy are difficult or impossible to detect using annual monitoring data.

Microcystins are usually considered as not having a role in the allelopathic processes [50], as the microcystin-producing cyanobacteria yield a number of other bioactive compounds. Nevertheless, microcystin produced by *Anabaena flos-aquae* caused mortality and lowered growth in chlorophyte *Chlamydonomas reinhardtii* [57], whereas in other studies the compound responsible for allelopathy in *Anabaena* has remained unknown [52,54,55]. In the present study, allelopathic effects of the filtrates did not correlate with their extracellular toxin concentration; thus it is unlikely that microcystin caused the observed reduction in *R*. *nottbecki* fluorescence. Further, it has been suggested that rather high pH is responsible for the observed allelopathic effect by cyanobacteria [58]. However, in the present study, pH of all filtrates used in the EC_50_ experiment was lower than that of a dense *R*. *nottbecki* culture (>9.7), indicating that elevation of culture pH to a level intolerable for *R*. *nottbecki* by the *Dolichospermum* filtrates did not cause the observed allelopathic effects.

Summertime surface water salinity showed a negative trend in the western Gulf of Finland and northern Baltic Proper, based on long-term monitoring data collected between 1979 and 2013. The result was expected as salinity started to decline already in the beginning of the 1980s [5,6,7]. The salinity decrease is largely due to larger inflow of freshwater following increasing climate-related precipitation [1]. In addition, saline water inflows to the Baltic Sea via the Danish Straits from the North Sea are not as common today as before; the inflows are expected to drop also in the future [59], as a consequence of mild winters and positive NAO indices [60]. At the same time, *Dolichospermum* biomass showed an increasing trend. This trend has not been reported before as phytoplankton trends in the Baltic Sea have usually been analysed at the class-level. Previous trend analyses [6,7] have shown increasing late-summer biomasses for cyanobacteria in the northern Baltic Proper and the Gulf of Finland, but not in the southern Baltic Sea [34]. The main factors affecting the increase of filamentous, diazotrophic cyanobacteria, including *Dolichospermum*, are warmer surface waters, rising phosphate levels, and decreasing salinity [7]. In the current study we demonstrate that on a salinity scale 0–6, the growth rate of *Dolichospermum* spp. is highest in freshwater. Further, the allelopathic properties of *Dolichospermum* spp. seem to be enhanced in intermediate salinities, to be lowest in freshwater, and cellular microcystin concentration is reduced in the lower salinity range. With decreasing salinities the habitat for *Dolichospermum* is getting more favourable especially in the northern and eastern parts of the Baltic Sea. Thus, the species complex will probably get more common in the future, perhaps at the extent of brackish water species, such as *N*. *spumigena*.
